# Sustained Mpox Proctitis with Primary Syphilis and HIV Seroconversion, Australia

**DOI:** 10.3201/eid2903.221845

**Published:** 2023-03

**Authors:** Rachel M. Burdon, David Atefi, Jainoor Rana, Arun Parasuraman, Andie S. Lee, Blake Nield

**Affiliations:** Sydney Local Health District, Sydney, New South Wales, Australia (R.M. Burdon, D. Atefi, J. Rana, A. Parasuraman);; Royal Prince Alfred Hospital, Sydney (A.S. Lee, B. Nield)

**Keywords:** Monkeypox virus, mpox, monkeypox, syphilis, acute HIV seroconversion, proctitis, sexually transmitted infections, Australia, HIV/AIDS, viruses

## Abstract

A 26-year-old man in Australia who has sex with men had severe perianal ulceration, proctitis, and skin lesions develop. Testing revealed primary syphilis, mpox, and primary HIV infection. Recent publications have documented severe mpox associated with HIV infection. Disruption of mucosal integrity by mpox lesions could enable HIV transmission and vice versa.

Human mpox (formerly monkeypox) is a viral zoonosis caused by monkeypox virus (MPXV). As of December 8, 2022, a total of 144 reported infections had occurred in Australia, all in men who have sex with men (MSM); no mpox-related deaths had been reported.

A 26-year-old overseas-born MSM with no comorbidities sought care at the Sydney Local Health District (SLHD; Sydney, NSW, Australia) Department of Sexual Health Medicine (DSHM), 20 days after becoming unwell with severe perianal ulceration, dyschezia, tenesmus, purulent bloody anal discharge, and skin lesions on his trunk and limbs. His symptoms were initially accompanied by a fever and prodrome, although those had resolved by the time he was examined. He had not received MPXV vaccination and had not taken HIV preexposure prophylaxis.

The patient spoke limited English and had traveled to Australia approximately 3 weeks earlier. He sought care from a local doctor 5 days after onset of symptoms and was prescribed valaciclovir for a clinical diagnosis of herpes simplex virus (HSV). He was unresponsive to treatment and returned to the same doctor 2 weeks later with more extensive perianal lesions and rectal symptoms and new truncal and limb lesions. This doctor contacted the SLHD Public Health Unit, which recommended referral to SLHD DSHM.

Physical examination showed large, superficial, ulcerated lesions with surrounding erythema perianally extending to the buttocks and 2 smaller, deep, indurated ulcers on the anal verge consistent with syphilitic chancres. The patient had purulent discharge from the anus; anoscopy was not attempted because of pain. Several umbilicated, papular lesions with necrotic centers and surrounding erythema were present on the hand, thigh, and trunk. The papular and perianal lesions were swabbed for MPXV, HSV, and syphilis PCR. We performed testing for HIV, chlamydia, and gonorrhea on urine, throat, and rectal specimens and took additional rectal swab specimens for PCR testing for lymphogranuloma venereum, syphilis, HSV, and *Mycoplasma genitalium*. He was treated with 4.8 million units of benzathine benzylpenicillin for presumptive primary syphilis and asked to isolate at home until MPXV results were known.

The patient reported his most recent sexual activity (receptive anal intercourse using a condom) was with a casual partner overseas 7–10 days before his arrival in Australia. The next most recent sexual encounter was 3 months earlier with his regular male partner of 1 year. He had not been to any nightclubs, sex-on-premises venues, or festivals and reported no other intimate contact. His last sexual health screening (including an HIV test) 7 months before detected no sexually transmitted infections.

Results of an HIV antigen/antibody assay were reactive (signal-to-cutoff ratio of 74.2 and a positive p24 antigen), whereas Western blot results were indeterminate (positive band at p24 and gp160), consistent with seroconversion. MPXV was detected at all sites by PCR, and results of syphilis enzyme immunoassay and *Treponema pallidum* particle agglutination assays were positive (rapid plasma reagin test result of 16). Syphilis PCR results were negative, but clinical signs and serologic testing were consistent with primary syphilis. Test results for gonorrhoea, chlamydia, *Mycoplasma genitalium*, HSV, varicella zoster, and hepatitis B and C were all negative.

We began antiretroviral treatment (bictegravir/emtricitabine/tenofovir alafenamide) in anticipation that immune reconstitution would improve the patient’s severe and sustained mpox (*1*). His rectal symptoms and perianal lesions improved dramatically after benzylpenicillin administration, and he began oral cefalexin for empirical treatment of secondarily infected mpox lesions. His HIV viral load was found to be 7,120,000 RNA copies/mL and CD4 count was 370 × 10^6^ cells/L (12%) with a fully sensitive genotype. He continued to improve and was deisolated once his skin lesions had resolved.

Severe and protracted mpox infection in persons living with HIV has been described previously (*2*–*5*). Publications have reported mpox co-occurring with HIV (*5*–*7*), syphilis (*8*), COVID-19 (*9*), and varicella zoster (*10*) in up to 15% of mpox cases (*3*). Boesecke et al. (*6*) report a case of severe mpox in the setting of advanced HIV and syphilis. However, no case reports have described mpox in the context of primary syphilis and primary HIV infection. The incubation periods for mpox, HIV infection, and syphilis aligned with the patient’s symptom onset 13 days after his only sexual encounter in the preceding 3 months, suggest co-infection likely acquired from a single encounter (Figure). This finding could indicate increased transmissibility of >1 infection from a partner with multiple infections, especially if the person had active syphilis or mpox skin lesions. Brundu et al. (*5*) postulated a disruption of mucosal integrity by mpox lesions could enable HIV transmission and that HIV infection also enables mpox acquisition. Previous publications have hypothesized local inoculation of the virus aggravated by an immune system dysfunction in the setting of acute HIV infection as a potential mechanism. HIV can result in atypical clinical manifestations of mpox and higher rates of secondary bacterial infections (*2*,*6*,*7*,*9*), consistent with this case-patient’s clinical course. This case highlights the need for comprehensive clinical assessment, a broad differential diagnosis, and syndromic testing for MPXV when evaluating patients with anogenital lesions.

**Figure:**
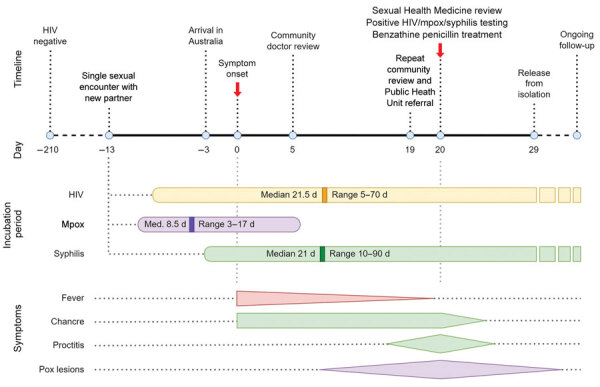
Timeline of symptom development and infectious vector incubation periods in case of patient with sustained mpox proctitis with primary syphilis and HIV seroconversion, Australia
